# Topographic Organization of Hippocampal Inputs to the Anterior Olfactory Nucleus

**DOI:** 10.3389/fnana.2018.00012

**Published:** 2018-02-22

**Authors:** Afif J. Aqrabawi, Jun Chul Kim

**Affiliations:** ^1^Department of Cell & Systems Biology, University of Toronto, Toronto, ON, Canada; ^2^Department of Psychology, University of Toronto, Toronto, ON, Canada

**Keywords:** AON, tracing, topography, hippocampus, olfaction, anterograde, retrograde, CA1

## Abstract

Top-down processes conveying contextual information play a major role in shaping odor representations within the olfactory system, yet the underlying mechanisms are poorly understood. The hippocampus (HPC) is a major source of olfactory top-down modulation, providing direct excitatory inputs to the anterior olfactory nucleus (AON). However, HPC-AON projections remain uncharacterized. In an effort to understand how hippocampal inputs are distributed within the AON, we systematically outlined their organization using anterograde and retrograde tracing methods. We found that AON-projecting hippocampal pyramidal neurons are located mostly in the ventral two-thirds of the HPC and are organized topographically such that cells with a ventral to intermediate hippocampal point of origin terminate, respectively, at the medial to lateral AON. Our neuroanatomical findings suggest a potential role for the HPC in the early processing and contextualization of odors which merits further investigation.

## Introduction

Olfaction is the primary sensory modality employed to perceive chemical signals in the environment. Within the olfactory bulb (OB), neural input arriving from olfactory sensory neurons terminate in glomeruli onto the dendrites of mitral and tufted cells (Vassar et al., 1994; Shepherd et al., 2004). The axons of these neurons fasciculate and form the lateral olfactory tract, a fiber system which courses the ventrolateral aspect of the brain and innervates the olfactory and entorhinal cortex, among other regions (Haberly and Price, 1977; Nagayama et al., 2010). Each olfactory sensory neuron expresses only one type of odorant receptor (out of ~1000) and all cells which express a common receptor type terminate their axons within a unique glomerulus. This allows olfactory stimuli to be represented in a pattern of activity elicited at the glomeruli termed an “odor map” (Ressler et al., 1994; Mombaerts et al., 1996). The neural representation is then processed further downstream where it is ultimately reconstructed into a perceived “odor object” (Wilson and Sullivan, 2011).

Although olfactory perception is largely shaped by bottom-up inputs received by the OB, it is also modulated by top-down inputs based upon prediction, expectation and memory of previous experiences (Freeman and Schneider, 1982; Kay and Laurent, 1999; Doucette and Restrepo, 2008; Mandairon and Linster, 2009; Moreno et al., 2009). Thus, the neural and behavioral responses to a given odorant can vary over time for an individual and also among members of the same species. Such top-down control of olfaction is thought to occur by means of strong and diverse feedback connections arriving from higher cortical and limbic structures (Fletcher and Chen, 2010). The hippocampus (HPC), via its direct excitatory inputs to the olfactory system, is thought to be a major source of top-down modulation (Martin et al., 2007; Aqrabawi et al., 2016). However, the underlying neural mechanism remains obscure.

Central to understanding how the HPC influences olfaction is an appreciation of how hippocampal signals are organized within the olfactory system. Bulk labeling studies whereby axons of pyramidal cells are visualized with neuronal tracers have outlined a connection between the HPC and the olfactory cortex (Swanson and Cowan, 1977; de Olmos et al., 1978; Haberly and Price, 1978; Van Groen and Wyss, 1990; Cenquizca and Swanson, 2007). These studies revealed that hippocampal innervation of the olfactory system is most abundant in the anterior olfactory nucleus (AON), a ring-like cortical structure situated immediately posterior to the OB and anterior to the piriform cortex (Brunjes et al., 2005). However, these early efforts were limited in the scope of their investigation, addressing only the existence of the HPC-AON pathway. The present study is aimed at offering detailed insight into the anatomical connectivity between the HPC and the AON to better understand how hippocampal inputs influence olfaction.

In a series of anterograde and retrograde tracing experiments, we systematically examined the connectivity between the dorsoventral extent of the HPC and the medial, dorsal, and lateral aspects of the AON (mAON, dAON and lAON respectively). Our results demonstrate a topographic gradient in the organization of hippocampal outputs to the AON, such that a ventral-to-intermediate point of origin in the CA1/Subiculum resulted in a medial-to-lateral point of termination at the AON.

## Materials and Methods

### Animals

Forty-four 8–12 week old adult male C57Bl/6 mice (Charles River Laboratories, Quebec, QC, USA) were used. Prior to surgery, mice were group housed in a temperature-controlled room on a 12 h light/dark cycle with *ad libitum* access to food and water. All procedures were performed in accordance with the guidelines of the Canadian Council on Animal Care (CCAC) and the University of Toronto Animal Care Committee. Surgical procedures were performed on mice under general anesthesia with isoflurane and mounted onto a stereotaxic frame. The atlas of the mouse brain by Paxinos and Franklin (2007) was used to determine coordinates for guiding the stereotaxic infusion of tracers.

### Stereotaxic Surgery

For anterograde tracing experiments, AAV2/9-hSyn-hChR2(H134R)-eYFP (~10^12^ GC/ml) purchased from the Vector Core at the University of Pennsylvania and a total of 18 mice were used. Two groups of six mice received unilateral infusions of 0.3 μL of channelrhodopsin-2 (ChR2) into the dorsal HPC (no angle; two sets: AP: −2.18 ML: ±2.10 DV: −1.75, *n* = 3 or AP: −2.70 ML: ±2.20 DV: −2.00, *n* = 3) or ventral HPC (10° angle away from midline; AP: −2.92 ML: ±2.15 DV: −4.90, *n* = 6). One group of six mice received a simultaneous co-injection of AAV2/5-hSyn-hChR2-mCherry (~10^12^ GC/ml) and AAV2/5-hSyn-hChR2-eYFP into the dorsal (AP: −2.70 ML: ±2.20 DV: −2.00) and ventral HPC using a counterbalanced combination.

For the retrograde labeling experiments, a total of twenty four mice were used. Three groups of eight mice received a unilateral injection of 0.2 μL of red fluorescent retrobeads (LumaFluor Inc., Naples, FL, USA) into the mAON (10° angle toward midline; AP: +2.90 ML: ±1.10 DV: −3.42), dAON (no angle; two sets: AP: +2.80 ML: ±0.75 DV: −3.10, *n* = 4 or AP: +2.80 ML: ±1.5 DV: −3.10, *n* = 4), or lAON (no angle; AP: +3.20 ML: ±1.10 DV: −3.90), respectively. A lower injection volume was used compared to anterograde tracing experiments to limit spread of the infusion outside the boundaries of the AON and neighboring AON subregions. All infusions were made by means of pressure ejection at a rate of 0.1 μL/min through a cannula connected by Tygon tubing to a 10 μL Hamilton syringe (Hamilton, Reno, NV, USA) mounted onto an infusion pump. Following each infusion, the cannula was left in place for an additional 20 min before retraction to limit the spread of the tracer.

### Histology

Approximately 3 weeks following surgery mice were transcardially perfused with Phosphate-Buffered Saline (PBS, pH 7.4), followed by 4% paraformaldehyde in phosphate buffer. Brains were extracted and postfixed overnight at 4°C and subsequently cryoprotected with PBS containing 30% sucrose. Coronal 40 μm thick sections were obtained using a cryostat (Leica, Germany). The sections were slide-mounted, counterstained with 4′,6-diamidino-2-phenylindole (DAPI) for 10 min, and coverslipped with Aquamount (Polysciences Inc., Warrington, PA, USA). The wide-field fluorescent images were captured with a 4× objective on a fluorescent microscope (Olympus, Japan). eYFP and red fluorescent retrobeads signals were captured using an U-MWIBA3 filter cube (Ex460- 495, Em510-550, DM505) and an U-MWIG3 filter cube (Ex530-550, Em575IF, DM570), respectively. Confocal images were captured through a Quorum spinning disk confocal microscope (Zeiss) using a 20× objective lens and were subsequently analyzed with Volocity Software (Perkin Elmer). eYFP and red fluorescent retrobeads signals were excited with 491 and 561 nm laser, respectively. Adobe Photoshop CS6 (Adobe Systems Incorporated, San Jose, CA, USA) was used to adjust the brightness and contrast of representative sections.

### Cell Counting

Retrobead-positive cell counting was performed using the cellSens software (Olympus, Japan). The HPC was divided into three portions such that the dorsal one-third consisted of the anterior portion of the HPC prior to its transition into its longitudinal form in coronal sections (approximately 2.46 mm posterior to Bregma). The remaining ventral two-third of the HPC was further divided into intermediate and ventral HPC separated by the rhinal fissure (rf). Calibration parameters were established using randomly chosen tissue sections and were selected such that the threshold for cell diameter was high enough to exclude the majority of falsely identified cells (noise). Calibration parameters were maintained for consistent counting of all tissue sections. Tissue sections from three animals in each of the medial, dorsal, and lateral-AON injected groups were analyzed. The density of retrobead-positive cells found across nine coronal sections (number of labeled cells in region of interest/area of region) for each subject, and mean densities for each group were calculated.

## Results

### ChR2-YFP-mediated Anterograde Tracing Reveals the Topographic Organization of HPC Inputs to the AON

**Figure 1:**
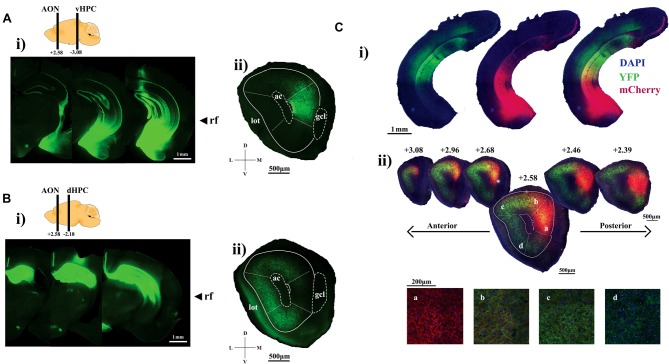
Visualizing hippocampal projection fibers at the anterior olfactory nucleus (AON). **(Ai)** Representative sections showing channelrhodopsin-2 (ChR2)-YFP expression within the ventral hippocampus (HPC). **(ii)** AON section illustrating ChR2-YFP-positive ventral HPC axon terminals tightly contained within the medial aspect. **(Bi)** Representative sections showing ChR2-YFP expression throughout the dorsal HPC. **(ii)** ChR2-YFP-positive dorsal HPC axon terminals preferentially target the dorsal, lateral and ventral parts of the AON. **(Ci)** AAV2/5-mediated expression of ChR2-YFP and ChR2-mCherry into the dorsal and ventral CA1, respectively. **(ii)** Representative sections of the AON illustrating hippocampal terminals forming a gradient as a result of partially overlapping patterns of ChR2-mCherry and YFP-positive axon terminals. Panels **(a–d)** represent confocal images of axon terminals found at the corresponding areas within the AON. The relative ratio of ChR2-mCherry and YFP-positive terminals gradually shifts along the medial to lateral aspects of the AON. Included are cartoon diagrams to illustrate A/P position of hippocampal terminals (left bar) and injection site (right bar) along with A/P coordinates relative to Bregma and arrows to indicate the longitudinal position of the rhinal fissure (rf). Ac, anterior commissure; lot, lateral olfactory tract; gcl, granule cell layer of the olfactory bulb (OB).

To examine the hippocampal innervation pattern of the AON, we first performed antero- grade tracing of hippocampal projections using virally delivered-ChR2 as an anterograde tracer (Gradinaru et al., 2009; Jennings and Stuber, 2015). AAV-hSyn-ChR2-YFP (ChR2 fused to eYFP driven by the human synapsin promoter) was infused into either the ventral (Figure 1Ai) or dorsal HPC (Figure 1Bi). Viral infusions targeting the ventral HPC led to expression of ChR2-YFP in the ventral half of the HPC below the rf, while dorsal HPC infusions led to ChR2-YFP expression in the dorsal half of the HPC above the rf (Figures 1Ai,Bi). In both cases, ChR2-YFP expression was restricted to the HPC with minimal spread into adjacent cortical structures such as the primary somatosensory cortex which does not project to the AON (Zakiewicz et al., 2014).

ChR2-YFP-positive axon fibers originating from the ventral half of the HPC were localized largely at the mAON with little signal observed in the OB, piriform cortex, or other AON subregions (Figure 1Aii). In contrast, projections from the dorsal half of the HPC were found predominantly in the dorsal, lateral, and ventral, but not medial, aspects of the AON, indicating a topographic organization of HPC inputs within the AON (Figure 1Bii). We confirmed this differential innervation pattern using two-color anterograde tracing by simultaneously expressing ChR2-YFP and ChR2-mCherry in the dorsal and ventral halves of the HPC, respectively (Figure 1Ci). Hippocampal projections established a gradient of innervation within the AON whereby YFP-positive and mCherry-positive HPC axon fibers partially overlapped at the junction between the dorsal and medial AON. The density of projections originating from the ventral half of the HPC gradually increased from the lateral to the medial AON whereas projections from the dorsal half of the HPC increased gradually from the medial to lateral AON (Figure 1Cii).

### Topographic Organization of HPC Inputs to the AON Is Further Confirmed by Retrograde Tracing

**Figure 2:**
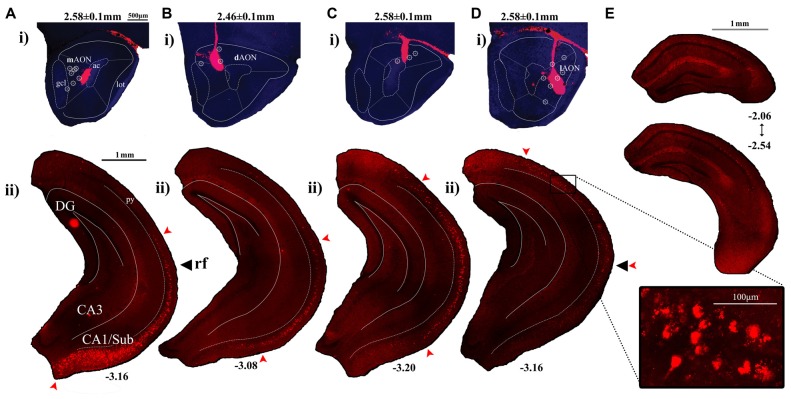
Labeling patterns observed in the CA1 following injections of red fluorescent retrobeads into the AON. **(Ai–Di)** Representative sections depicting retrobead injection sites at the AON and the resulting cell body labeling patterns within the HPC. Included are AP coordinates to indicate position of coronal sections relative to Bregma and arrows to indicate the longitudinal position of the rf. The superimposed symbol (۞) indicates central positions of the injection sites which have yielded similar labeling patterns. **(Aii)** Retrobead injections restricted to the medial aspect of the AON produced labeled cell bodies found in the ventral-most CA1/Subiculum, decreasing in a gradient which ends at the level of the rf. **(Bii)** Injections delivered into the dorsomedial region of the AON resulted in labeled cell bodies found at their greatest density along more dorsal positions within the intermediate CA1. Few labeled cells were observed in the ventral-most CA1/Subiculum. **(Cii)** Injections into the dorsal AON resulted in retrobead-labeled cell bodies at the level of the rf, decreasing in number towards both dorsal and ventral directions. **(Dii)** Injections which targeted further lateral positions within the AON resulted in labeled cell bodies found at their greatest density in the intermediate CA1, decreasing in numbers towards the ventral CA1. The boxed region depicts a confocal image of retrobead-labeled pyramidal cells captured using a 20× objective. **(E)** Dorsal hippocampal coronal sections indicate the range of AP positions relative to Bregma where cell bodies were first identified following injections into the lateral AON.

The ChR2-mediated anterograde tracing method cannot unambiguously distinguish hippocampal axon terminals from hippocampal fibers of passage at the AON. Furthermore, it remains unclear how the AON-projecting HPC cells are distributed along the dorso-ventral axis of the HPC. To overcome this limitation and further validate the topographic organization of hippocampal inputs, we performed retrograde tracing experiments by employing fluorescent retrogradely-transported microspheres (retrobeads) that are uptaken at presynaptic axon terminals. Retrobeads were microinjected unilaterally into the medial, dorsal, or lateral aspects of the AON in separate cohorts of mice (*n* = 4–8 for each group). The spread of injected retrobeads was restricted to the targeted AON subregions (Figures 2Ai–Di), which are denominated on the basis of their cardinal positions as previously described (Brunjes et al., 2005). In all cases, the majority of labeled cells were observed in the hemisphere ipsilateral to the injection site, with very few cells labeled in the contralateral HPC (data not shown).

Injection of retrobeads into the mAON yielded labeled cells found in their greatest density near the ventral-most part of the CA1/Subiculum subregion (Figures 2Aii, 3). These mAON-projecting cells in the ventral CA1 progressively decreased in number towards the dorsal end of the longitudinal axis, forming a gradient terminating at the level of the rf. In contrast, retrobead injections into the dAON produced the greatest density of labeled cells in the intermediate CA1 at or near the level of the rf, tapering gradually in both dorsal and ventral directions (Figures 2Bii,Cii, 3). Very few, if any, dAON-projecting cells were found at the ventral- and dorsal-most parts of the CA1. Finally, lAON injections produced retrobead-labeled cells which were found in their greatest density at the dorsal aspect of the intermediate CA1 and were progressively fewer towards the ventral end with the gradient terminating at the level of the rf (Figures 2Dii, 3). Notably, lAON injections yielded extremely sparse, if any, labeling in the dorsal one-third of the CA1 (Figure 2E); instead, labeled cells first appeared at the dorsal most extent of the ventral two-thirds of the CA1 (Bregma: ~ −2.06 to −2.54; Figure 2E), indicating that the dorsal CA1 is unlikely to represent a meaningful source of inputs to the AON. Taken together, our anterograde and retrograde tracing data revealed that hippocampal inputs originating from the ventral two-third of the HPC are topographically organized within the AON.

**Figure 3:**
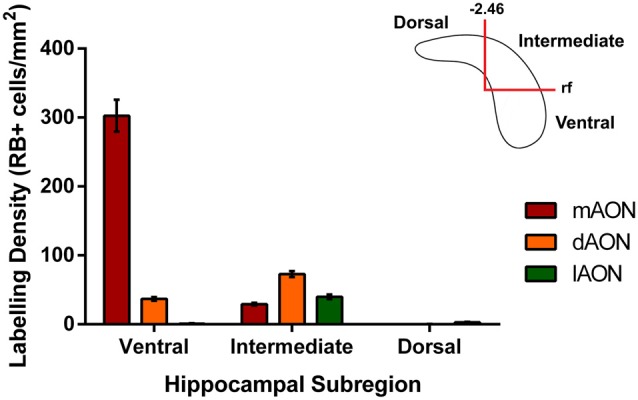
Quantification of retrobead-labeled cells in the HPC. Data illustrates average density of retrobead-labeled cells within different subregions of the CA1. Injections of retrobeads into the mAON produced the greatest density of labeled cells in the ventral HPC (302.7 ± 23.3 cells/mm^2^) with fewer cells present in the intermediate HPC (29.3 ± 2.0 cells/mm^2^). In contrast, dAON retrobead injections resulted in a greater density of cells in the intermediate HPC (72.7 ± 4.3 cells/mm^2^) than the ventral HPC (36.9 ± 2.6 cells/mm^2^). Injections in the lAON labeled cells found predominantly in the intermediate HPC (39.7 ± 3.3 cells/mm^2^) with virtually none found in the ventral (0.89 ± 0.5 cells/mm^2^) or dorsal (2.9 ± 0.7 cells/mm^2^) aspects. Top-right schematic depicts delineations made to produce the three hippocampal subregions based on anteroposterior position from Bregma and the rf.

## Discussion

**Figure 4:**
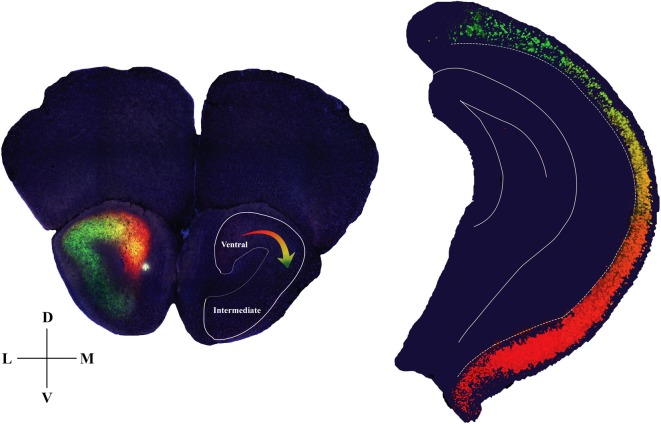
Diagram summarizing anatomical details of the hippocampal-AON pathway. Coronal section of the AON depicting CA1 pyramidal cell terminals in a gradient of innervation (left) which reflects the position of the cells (pseudo-colored from retrobead signals) within the hippocampal longitudinal axis (right). In the opposite hemisphere an outline is drawn to represent a hippocampal image that is created as a result of this innervation pattern.

The present study has revealed a topographic gradient in HPC-AON projections such that the ventral-most part of the HPC innervates most heavily the mAON, and progressively more dorsal parts of the HPC innervate increasingly more lateral positions at the AON as illustrated in our model (Figure 4). This pattern of connectivity suggests that the ventral two-third of the HPC superimposes an image of its entire dorsoventral axis onto the AON. This would also imply that the AON can be mapped in detail to reflect activity in an associated hippocampal area, in a manner analogous to the cortical homunculus representing a layout of the body.

Our tracing results are largely consistent with previous studies which examined the downstream targets of CA1 projection neurons (Swanson and Cowan, 1977; de Olmos et al., 1978; Haberly and Price, 1978; Van Groen and Wyss, 1990; Cenquizca and Swanson, 2007). AON-projecting hippocampal fibers join the white matter of the alveus and course through the fimbria-fornix to reach the lateral septum, nucleus accumbens, and the prefrontal cortex before they innervate the AON (Cenquizca and Swanson, 2007; Arszovszki et al., 2014). Alternatively, hippocampal fibers may also reach the AON via the longitudinal association bundle through which the HPC extends its axons to other sensory and visceral cortical areas. Our tracing methods do not distinguish these two routes, thus it remains to be determined to what extent each route contributes to the formation of the topographic innervation pattern observed at the AON.

The functional connection between the HPC and the olfactory system is growing increasingly apparent. The HPC has been implicated in different aspects of odor processing, including olfactory pattern separation, the formation of context-odor associations, coding of the temporal sequence of a series of odors, and olfactory learning and memory (Eichenbaum, 1998; Levy et al., 2004; Kent et al., 2007; Kesner et al., 2010, 2011; Weeden et al., 2014). Our anterograde tracing data demonstrate that hippocampal innervation of the olfactory system is largely restricted to the AON, suggesting that the AON plays a major role in mediating hippocampal modulation of olfaction. Further support for this idea is the AON’s optimal position as the initial recipient of input from the OB and the largest source of olfactory cortical feedback projections (Carson, 1984). The AON also maintains extensive connections to other cortical regions such as the entorhinal cortex, amygdala, hypothalamus and neuromodulatory centers, though little is known beyond its anatomical and electrophysiological properties (Haberly and Price, 1978; Brunjes et al., 2005).

The functional implication of the gradient in the HPC-AON connection remains unclear. Notably, a similar topographic organization is known to exist in other extrinsic targets of the HPC including the amygdala, nucleus accumbens, and lateral septum (Groenewegen et al., 1987; Risold and Swanson, 1997; Witter et al., 2000; Kishi et al., 2006). The HPC is well-documented to play a role in a variety of cognitive and emotional operations, among them are learning, memory, spatial navigation, anxiety and stress response-associated processing (for a review see Strange et al., 2014). Of note, a study by Dong et al. (2009) showed that the CA1 can be divided into three subregions based on the expression pattern of molecular spatial markers: dorsal (dCA1), intermediate (iCA1), and ventral (vCA1) CA1. The study found that the dCA1 occupies approximately dorsal one-third of the CA1 whereas iCA1 and vCA1 together occupy the ventral two-third of the CA1 (Dong et al., 2009) thereby providing a more precise organization scheme as an alternative to the often-arbitrary reference to dorsal and ventral HPC (Fanselow and Dong, 2010). Our results show that hippocampal inputs to the AON arise from the iCA1 and vCA1, but not dCA1, and that the iCA1 and vCA1 innervate the medial to lateral AON, respectively. The lack of feedback projections from the dCA1 is consistent with studies suggesting that odor information processing is restricted to the ventral CA1, yet this view remains controversial owing to recent findings which implicate olfactory involvement in dorsal HPC function, particularly in place field development (Kulvicius et al., 2008; Aikath et al., 2014; Igarashi et al., 2014; Zhang and Manahan-Vaughan, 2015). Thus, the functional role of the HPC-AON pathway can only be clear following further investigations into both structures.

Ultimately, the topographic organization of the HPC-AON pathway offers a unique model for investigating how information originating from distinct hippocampal subregions contribute to olfaction. In an initial effort to elucidate the role of hippocampal inputs to the AON, we recently showed that the vCA1-mAON pathway can modulate olfactory sensitivity (Aqrabawi et al., 2016). However, considering the well-established role of HPC in representing spatiotemporal context, it would be of great interest to investigate how iCA1 and vCA1 inputs to the AON differentially regulate olfactory memory.

## Author Contributions

AJA and JCK carried out the study conceptualization and experimental design and wrote the manuscript. AJA performed the surgical procedures.

## Conflict of Interest Statement

The authors declare that the research was conducted in the absence of any commercial or financial relationships that could be construed as a potential conflict of interest.
